# Supplementary material for: Isolation and Characterization of Neutralizing Monoclonal Antibodies from a Large Panel of Murine Antibodies against RBD of the SARS-CoV-2 Spike Protein
Source: Antibodies (Basel). 2024 Jan 5;13(1):5. doi: 10.3390/antib13010005 (PMC10801580; doi:10.3390/antib13010005)
Supplement: Supplementary file 1 [file antibodies-13-00005-s001.zip › antibodies-2747696-supplementary.pdf]

```
VH_mouse      EVQLQQSGAELVKPGASVKLSCTASGFNIKETYVHWVKQRPEQGLEWIGRIDHAIGDSEYDPKFQGKATVTADTSSN
VH1           QVQLVQSGAEVKKPGASVKVSCKASGFNIKETYVHWVRQAPGQGLEWMGRIDHAIGDSEYAQKFQGRVTMTRDTSIS
VH2           QVQLVQSGAEVKKPGASVKVSCKASGFNFKETYMHWVRQAPGQGLEWMGRIDHAIGDSEYAQKFQGRVTMTRDTSIS
VH3           QVQLVQSGAEVKKPGASVKVSCKASGFNIKETYVHWVRQAPGQGLEWMGRIDPAIGDSEYAQKFQGRVTMTRDTSIS
VH4           QVQLVQSGAEVKKPGASVKVSCKASGFNIKETYVHWVRQAPGQGLEWMGRIDHAIGDSEYDPKFQGRVTVTADTSIS
VH5           QVQLVQSGAEVKKPGASVKVSCKASGFNIKETYVHWVRQRPEQGLEWMGRIDHAIGDSEYDPKFQGRVTVTADTSSN
humVH_germl   QVQLVQSGAEVKKPGASVKVSCKASGYTFTGYYMHWVRQAPGQGLEWMGRINPNSGGTNYAQKFQGRVTMTRDTSIS

VH_mouse      TAYLQLSRLTSEDTAVYYCARTWGPFFDFWGQGTTVTVSS
VH1           TAYMELSRLRSDDTAVYYCARTWGPFFDFWGQGTLVTVSS
VH2           TAYMELSRLRSDDTAVYYCARTWGPFFDFWGQGTLVTVSS
VH3           TAYMELSRLRSDDTAVYYCARTWGPFFDFWGQGTLVTVSS
VH4           TAYMELSRLRSDDTAVYYCARTWGPFFDFWGQGTLVTVSS
VH5           TAYMELSRLRSDDTAVYYCARTWGPFFDFWGQGTLVTVSS
hum_VH_germl  TAYMELSRLRSDDTAVYYCAR    YFDYWGQGTLVTVSS


VL_mouse      DVVMTQTPLSLPVSLGDQASISCRSSQSLVHSHGNTFLHWYLQKPGQSPKVLIYKVSSRFSGVPDRFSGSGAGT
VL1           DIVMTQTPLSLSVTPGQPASISCKSSQSLLHSHGNTFLYWYLQKPGQSPQLLIYKVSSRFSGVPDRFSGSGSGT
VL2           DIVMTQTPLSLSVTPGQPASISCKSSQSLVHSHGNTFLHWYLQKPGQSPQLLIYKVSSRFSGVPDRFSGSGSGT
VL3           DIVMTQTPLSLSVTPGQPASISCKSSQSLLHSHGNTFLHWYLQKPGQSPQVLIYKVSSRFSGVPDRFSGSGSGT
VL4           DIVMTQTPLSLSVTPGQPASISCKSSQSLVHSHGNTFLHWYLQKPGQSPKVLIYKVSSRFSGVPDRFSGSGAGT
humVL_germl   DIVMTQTPLSLSVTPGQPASISCKSSQSLLHSDGKTYLYWYLQKPGQSPQLLIYEVSSRFSGVPDRFSGSGSGT

VL_mouse      DFTLKISRVEAEDLGVYFCSQSTHVPYTFGGGTKLEIK
VL1           DFTLKISRVEAEDVGVYYCSQSTHVPYTFGQGTKLEIK
VL2           DFTLKISRVEAEDVGVYYCSQSTHVPYTFGQGTKLEIK
VL3           DFTLKISRVEAEDVGVYYCSQSTHVPYTFGQGTKLEIK
VL4           DFTLKISRVEAEDVGVYFCSQGTHVPYTFGGGTKLEIK
humVL_germl   DFTLKISRVEAEDVGVYYCMQGIHLPYTFGQGTKLEIK
```

Figure S1. **Humanization process of VH and VL chains**. Humanized VH (top) and VL (bottom) variable domains. Mutations relative to the closest murine germline (IGHV14-3*02 IGHJ2*01, IGKV1-110*01 IGKJ2*01) are indicated in red. Mutations in the humanized variable domains relative to the human germline sequence (IGHV1-2*06 IGHJ4*01, IGKV2-29*02 IGKJ2*01) are indicated in blue.

In vivo 9-8F2-B11 efficacy in K18-hACE2 mice

The efficacy study was conducted on K18-hACE2 mice, immunocompetent transgenic mice expressing the human form of the ACE2 receptor under the control of the human keratin 18 promoter.

At day 0 the mice (N=3) were anaesthetised by inhalation of isofluorane and infected intranasally with 10 µl/narice of Pseudovirus SARS-CoV2-Spike (D614G)-Luc or with a Lentivirus-Luc used as a negative control for infection (N=3). 2 hours before and 4 hours after infection, the animals were treated with 10 µl/narice of anti-SARS-CoV2 monoclonal antibody 9-8F2-B11 or with PBS (control group for efficacy).

During the course of the study, the clinical signs of the animals were evaluated and their body weight was recorded daily. Since Pseudovirus carries the gene for luciferase, it was possible to monitor the infection by acquisition of the bioluminescence signal produced.

At 48-, 72- and 96-hours post-infection, all animals were analysed using the IVIS 200 Imaging System in order to quantitatively measure the bioluminescence associated with the presence of the pseudo virus.

At 96 hours after infection, after *in vivo* analysis, the animals were sacrificed by asphyxiation and the nasal turbinates and lungs were removed from each.

The explanted organs were also analysed ex vivo by IVIS for evaluation of associated bioluminescence.

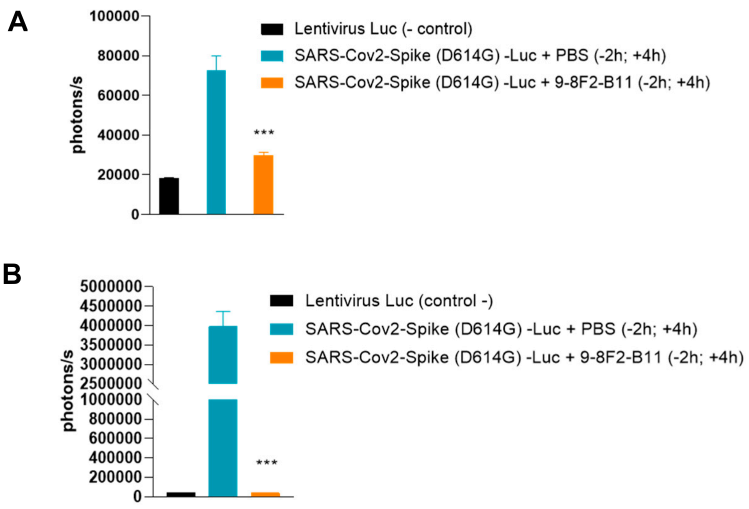

Figure S2. 9-8F2-B11 shows high efficacy in neutralizing SARS-CoV-2 in K18-hACE2 mice. The two graphs show mean values ±SD (photons/s) of the biolumiscence detected in K18-hACE2 mice in both the total body of the animal (**A**) and the turbinates (**B**) at 96h after infection.
